# Analysis of Mutation Rate of 17 Y-Chromosome Short Tandem Repeats Loci Using Tanzanian Father-Son Paired Samples

**DOI:** 10.1155/2018/8090469

**Published:** 2018-08-06

**Authors:** Fidelis Charles Bugoye, Elias Mulima, Gerald Misinzo

**Affiliations:** ^1^Department of Forensic Science and DNA Services, Government Chemist Laboratory Authority, Dar es Salaam, Tanzania; ^2^Department of Veterinary Microbiology, Parasitology and Biotechnology, Sokoine University of Agriculture, Morogoro, Tanzania

## Abstract

Hundred unrelated father-son buccal swab sample pairs collected from consented Tanzanian population were examined to establish mutation rates using 17 Y-STRs loci DYS19, DYS389I, DYS389II, DYS390, DYS391, DYS392, DYS393, DYS385a, DYS385b, DYS437, DYS438, DYS439, DYS448, DYS456, DYS458, DYS635, and Y-GATA-H4 of the AmpFlSTRYfiler kit used in forensics and paternity testing. Prior to 17 Y-STRs analysis, father-son pair biological relationships were confirmed using 15 autosomal STRs markers and found to be paternally related. A total of four single repeat mutational events were observed between father and sons. Two mutations resulted in the gain of a repeat and the other two resulted in a loss of a repeat in the son. All observed mutations occurred at tetranucleotide loci DYS389II, DYS385a, and DYS385b. The locus specific mutation rate varied between 0 and 1.176 x10^−3^ and the average mutation rate of 17Y-STRs loci in the present study was 2.353x10^−3^ (6.41x10^−4^ - 6.013x10^−3^) at 95% CI. Furthermore the mean fathers' age with at least one mutation at son's birth was 32 years with standard error of 2.387 while the average age of all fathers without mutation in a sampled population at son's birth was 26.781 years with standard error of 0.609. The results shows that fathers' age at son's birth may have an effect on Y-STRs mutation rate analysis, though this age difference was statistically not significant using unpaired samples t-test (p = 0.05). As a consequence of observed mutation rates in this study, the precise and reliable understanding of mutation rate at Y-chromosome STR loci is necessary for a correct evaluation and interpretation of DNA typing results in forensics and paternity testing involving males. The criterion for exclusion in paternity testing should be defined, so that an exclusion from paternity has to be based on exclusion constellations at a minimum of two 17 Y-STRs loci.

## 1. Introduction

Research and application of Y-chromosome short tandem repeats (Y-STRs) have proven beneficial in a number of fields including paternity, anthropology, and genealogical studies [1]. The very useful application of Y-STR systems is due to their potential in detecting and discriminating male DNA. Human Y-STR polymorphisms or microsatellites are useful in resolving and relating male lineages in forensics especially in sexual assault cases where there is a large proportion of mixed male/female stains [2], genealogical [3], evolutionary studies [4], and anthropological applications [5].

The interpretation of DNA evidence in forensic analysis and paternity testing is based on the similarities or differences at a genetic loci used. In parenthood testing, the difference at inheritable genetic marker loci between the putative father and the offspring is attributed to nonbiological paternity and therefore leads to exclusion of biological paternity. On the other hand, the spontaneous mutations in the germline of the putative father at any genetic marker locus used in the analysis can lead to an erroneous exclusion because such mutation results in differences between the parent and offspring. Since new alleles occur due to the mutation events, there is natural correlation between the degree of polymorphism and the underlined mutations rate of any given locus; i.e., the higher the mutation rate is, the more variable the locus is [6].

In forensic DNA typing applications, highly polymorphic loci are usually preferred due to their high power of discrimination. Therefore, short tandem repeat (STR) loci or microsatellites are considered to be the markers of choice in forensics because of their high power of discrimination and ease of analysis. For criminal and paternity testing investigations, which involve males with deceased alleged father, Y-STRs are used as the marker of choice [7]. Y-STRs are preferred because they are transmitted without recombination from fathers to sons and therefore are able to characterise paternal pedigree. In addition, Y-STRs are suitable for sexual assault investigations as they provide male specific DNA profiles which avoid problems of mixed stain interpretation [1]. However, since highly polymorphic Y-STR loci applied in forensic investigations constantly evolve through mutations, the evaluation and interpretation of the genetic profiles requires precise knowledge on mutation rates at each loci used. Reliable estimations of mutation rates for these loci are valuable in assisting the interpretation of Y-STRs test results. Most of investigations have reported mutation rates for the minimal haplotype loci in different populations, but very few articles have reported results with the 17 Y-STRs loci mutation rates using African populations.

## 2. Materials and Methods

During this study, buccal swab samples were collected from consented father-son paired samples whose biological relationship was confirmed by autosomal STRs using AmpFlSTR Identifiler kit [8]. A total of 100 father-son pairs from Tanzanian population were collected in Dar es Salaam after obtaining informed consent for participation in the study. DNA extraction was done using Chelex method and the extracted DNA were amplified using 17Y-STRs of AmpFlSTRYfiler™ kit (DYS456, DYS389I, DYS390, DYS389II, DYS458, DYS19, DYS385a, DYS385b, DYS393, DYS391, DYS439, DYS635, DYS392, Y-GATA-H4, DYS437, DYS438, and DYS448) [8] using the following conditions: PCR amplification of the Y filer loci was performed using 0.5–1 ng of DNA template and the total 25 *μ*L reaction volumes of PCR amplification were used, as recommended by the manufacturer. The PCR amplicons were analyzed using capillary electrophoresis in an ABI Prism 3130xl genetic analyser [8]. Analysis of DNA fragments was performed using a Gene Mapper IDv.3.2 [8]. Data analysis was carried out using the Excel statistical Software [9], and the confidence interval (CI) was estimated from the binomial standard deviation [10].

## 3. Results and Discussion

### 3.1. 17 Y-STRs Locus Specific Mutation Characteristics

Analysis of locus specific mutation characteristics using 17 Y-STRs loci in Tanzanian father-son pairs of DNA confirmed biological paternity revealed four mutations events which were identified on DYS385a, DYS385b, and DYS389II among 17Y-STRs loci analyzed [9] (Table 1).

However, no mutation event was observed for DYS19, DYS389I, DYS390, DYS391, DYS392, DYS393, DYS437, DYS348, DYS439, DYS448, DYS456, DYS458, DYS635, and Y-GATA-H4 loci analyzed. The observed locus specific mutation rate ranged between 0 for DYS19, DYS389I, DYS390, DYS391, DYS392, DYS393, DYS437, DYS348, DYS439, DYS448, DYS456, DYS458, DYS635, and Y-GATA-H4 loci and 1.765 × 10^−3^ (1.43x10^−4^– 4.243x10^−3^) for DYS385a locus at 95% CI [9]. Among 100 father-son pairs analyzed at the same 17 Y-STRs loci, there was no observation of multiple Y-chromosome microsatellite mutation within the same germline transmission or nonuniform alleles such as microvariants, duplication, and triplication that have been previously reported by Laouina [11] in Moroccan population.

The highly polymorphic Y-STR locus DYS385 was observed to have a higher mutation rate compared to all other Y-STRs loci analyzed (Table 2). In this study, the observed higher specific locus mutation rate for Y-STR locus DYS385a/b (if treated as single locus) was 1.765 × 10^−3^ followed by mutation rate of 5.88x10^−4^ for locus 389II [9].

All observed mutation events were characterised by single-step mutations (Table 1), in accordance with the generally accepted mutation model for microsatellites, in which the alleles are known to mutate primarily through the gain and loss of single repeat units [12, 13].

In addition, two tetranucleotide microsatellites loci DYS385 and 389II appeared to consist of higher average mutation rates among all 17 Y-STRs analyzed compared to all other trinucleotide and dinucleotide microsatellite loci [9]. Similar locus specific mutation characteristics were found in Moroccan's population in a sample of 252 father-son pairs using 17 Y-STRs loci by Laouina [11] in which average locus specific mutation rate was higher at tetra nucleotide microsatellites loci. This higher mutation rate on tetranucleotide microsatellites was also observed by Kayser et al. [1] using 15 Y-STRs loci for a total of 4999 male germline transmission from father-son pairs of previously confirmed paternity. Furthermore, the single loss mutation characteristics event observed in this study was in agreement with research results found by Farfán and Prieto [14] where three single-step loss mutations were observed at DYS389II loci, during mutations analysis at 17 Y-STR loci in father-son pairs from southern Spain.

### 3.2. Analysis of 17Y-STRs Locus Specific Mutation Rate

Using 100 Tanzanian father-son paired samples with confirmed paternity covering 1700 meioses were used to estimate 17Y-STRs locus specific mutation rate. The observed average estimates of 17 Y-STRs locus specific mutation rate ranged from 0 to 1.765 × 10^−3^ (1.43x10^−4^ - 4.243x10^−3^) at 95% CI. The higher average locus specific mutation rate was found at DYS385a locus while mutation rates of 5.88x10^−4^ (1.5x10^−5^ – 3.273x10^−3^) at 95% CI were observed for both DYS385b and DYS389II loci. There was no mutation observed for DYS19, DYS389I, DYS390, DYS391, DYS392, DYS393, DYS437, DYS348, DYS439, DYS448, DYS456, DYS458, DYS635, and Y-GATA-H4 loci analyzed [9].

The average mutation rate across all markers in this study was 2.353x10^−3^(6.41 × 10^−4^ - 6.013x10^−3^) at 95% CI [9] (Table 2). This overall average mutation rate is nearly similar to those reported by Sanchez-Diz [15] in which a mutation rate of 2.2 × 10^−3^ in 701 father-son pairs in Iberian and Latin America groups was found. Dupuy et al. [16] used 1766 father-son pairs to analyze 9Y-STRs alone in Norway's population and found the average mutation rate of 2.3x10^−3^. A similar mutation rate of 2.3x10^−3^ was found by Lee et al. [17] in collection of Y-STRs mutation events for Korean population using a high number of loci, 22 Y-STRs in a sample size equal to 369 father-son pairs. Furthermore, the average mutation rate estimated in this study is not significantly different from the average mutation rate of autosomal STR loci commonly used in forensics as previously reported from family analysis. In their findings, the autosomal STRs mutation rate of 2.1 × 10^−3^ was reported by Brinkman et al., 1998; mutation rate of 2.7 × 10^−3^ was reported by Henke and Henke [18] whereas mutation rate of 0.6 × 10^−3^ was reported by Sajantila [19].

The average mutation rate for 17Y-STRs loci found in this research study is greater than those calculated by Viera-Silva [20] in a sample of 95 father-son pairs from Portugal (1.85 × 10^−3^) and by Farfán and Prieto [14] using 17 Y-STRs loci from southern Spain population was 1.563 × 10^−3^ (0.322 × 10^−3^ - 4.559 × 10^−3^) at 95% CI, but the average mutation rate found in this study is less than those calculated by Decker [21] for Caucasian and Asians populations cited up; in US admixed sample of 399 father-son pairs using 17 Y-STRs, the mutation rate found was 3.13x10^−3^.

The results of present study are in general agreement with the fore mentioned research findings in which all the same 17 Y-STRs set or other number Y-STRs loci used the average mutation rate observed were in the order of 10^−3^ though number of father-son pairs varied between the mentioned studies above. Since there were no significant differences in mutation rate observed, therefore the mutation rates analysis does not depend on the sample size or number of Y-STRs loci used but population diversity [9].

### 3.3. Effects of Father's Age on 17 Y-STR Mutation Rate Analysis

The present study shows that the average fathers' age with at least one mutation at son's birth was 32 years with standard error of 2.387 while the average age of all fathers without mutation in a sampled population at son's birth was 26.781 years with standard error of 0.609 (Table 3). Results shows undoubtedly the age of the mutated father from our study which is marginally older than that without mutations. The results clearly shows that fathers' age at son's birth may have an effect on Y-STRs mutation rate analysis, though this age difference is statistically not significant using unpaired samples t-test (p = 0.05) [9]. The results of present study are in agreement with results of research findings by Sanchez-Diz [15], Lee [17], and Goebloed [22] who also reported relatively older average age of mutated fathers in their studies but the age difference between fathers' age with at least one mutation and fathers' age without mutation was found statistically not significant.

## 4. Conclusion

The results of 17-Y-STRs mutation observed from this study revealed that the precise and reliable understanding of mutation rate at Y-chromosome short tandem repeats loci is necessary for a correct evaluation and interpretation of DNA typing results in forensics and paternity testing involving males. Based on the findings, the criterion for exclusion in paternity testing should be defined in any DNA testing laboratory using 17-Yfiler Amplification kits, so that an exclusion from paternity has to be based on exclusion constellations at the minimum of two 17 Y-STRs loci.

## Figures and Tables

**Table 1:** Mutation count and Y-STRs loci mutation characteristics events as revealed by direct observation on father-son paired samples of previously confirmed biological relationship.

Sample ID's	Loci	Repeat sequence^a^	Father's profile	Son's profile	Mutation characteristics	Mutation count
F/C003	DYS385b	GAAA	16	17	Gain	1
F/C012	DYS385a	AAGG	15	16	Gain	1
F/C074	DYS385a	AAGG	16	15	Loss	1
F/C082	DYS389II	CTGT/CTAT	31	30	Loss	1

^a^Repetitive sequence structure previously reported by Gusmao and Carracedo (2003).

**Table 2:** Mutation count, mutation rate and 95% confidence interval (CI) for the 17 Y-STRs loci studied using Tanzanian father-son paired samples.

**Loci**	**Repetitive DNA sequence** ^**a**^	**Mutation count**	**Allele transmission**	**Mutation rate**	** 95**%** CI**
DYS19	CTAT/CTAC	0	1700	0.000	0.000 – 2.168x10^−3^
DYS389I	AAGG/GAAA	0	1700	0.000	0.000 – 2.168x10^−3^
DYS389II	CTGT/CTAT	1	1700	5.88x10^−4^	1.5x10^−5^ – 3.273x10^−3^
DYS390	CTGT/CTAT	0	1700	0.000	0.000 – 2.168x10^−3^
DYS391	CTGT/CTAT	0	1700	0.000	0.000 – 2.168x10^−3^
DYS392	ATT	0	1700	0.000	0.000 – 2.168x10^−3^
DYS393	GATA	0	1700	0.000	0.000 – 2.168x10^−3^
DYS385a	AAGG	2	1700	1.176x10^−3^	1.43x10^−4^– 4.243x10^−3^
DYS385b	GAAA	1	1700	5.88x10^−4^	1.5x10^−5^ – 3.273x10^−3^
DYS438	TTTTC/TTTTA	0	1700	0.000	0.000 – 2.168x10^−3^
DYS439	GATA	0	1700	0.000	0.000 – 2.168x10^−3^
DYS437	TCTA/TCTG	0	1700	0.000	0.000 – 2.168x10^−3^
DYS448	AGAGAT	0	1700	0.000	0.000 – 2.168x10^−3^
DYS458	GAAA	0	1700	0.000	0.000 – 2.168x10^−3^
DYS456	AGAT	0	1700	0.000	0.000 – 2.168x10^−3^
DYS635	TCTA/TGTA	0	1700	0.000	0.000 – 2.168x10^−3^
Y GATA H4	TAGA	0	1700	0.000	0.000 – 2.168x10^−3^
**Average**		**4**	** 1 700**	**2.353x10** ^-**3**^	**6.41x10** ^-**4 **^ **- 6.013x10** ^-**3**^

**Table 3:** Fathers's age at the time of Sons' birth with at least one mutation and without any mutation on 17 Y-STRs loci.

**Fathers' age (Years) with at least one mutation**	**Fathers' age (Years) without any mutation**
25,40,32,31	19,20,26,25,30,21,25,26,24,30,32,30,21,28,30,30,24,25,34,20
	35,18,19,18,19,20,32,36,32,33,34,32,21,20,25,34,29,28,35,25
	24,26,25,24,26,25,34,23,25,24,28,29,25,26,23,24,27,28,36,26
	37,25,34,20,34,18,19,18,19,39,32,33,32,33,36,32,42,34,20,18
	18,19,26,19,20,32,33,32,33,36,25,20,24,18,29,19

**Average = 32.000**	**Average = 26.781**
**Standard error = 2.387**	**Standard error = 0.609**
**P Value = 0.060**	

## Data Availability

The mutation data used to support the findings of this study are included within the articles. The generated Sons' Y-STRs haplotype data were only submitted to YHRD (http://www.yhrd.org/) and received the Accession no. YC000312.
